# Supplementary material for: Relationship between Renal Function, Fibrin Clot Properties and Lipoproteins in Anticoagulated Patients with Atrial Fibrillation
Source: Biomedicines. 2022 Sep 13;10(9):2270. doi: 10.3390/biomedicines10092270 (PMC9496227; doi:10.3390/biomedicines10092270)
Supplement: Supplementary file 1 [file biomedicines-10-02270-s001.zip › biomedicines-1896805-supplementary.pdf]

Relationship between Renal Function, Fibrin Clot Properties and Lipoproteins in Anticoagulated Patients with Atrial Fibrillation

Wern Yew Ding [1,*], Ian G. Davies [2], Dhiraj Gupta [1] and Gregory Y. H. Lip [1,3]

1 Liverpool Centre for Cardiovascular Science, University of Liverpool and Liverpool Heart & Chest Hospital, Liverpool L14 3PE, UK
2 Research Institute of Sport and Exercise Science, Liverpool John Moores University, Liverpool L3 5UX, UK
3 Department of Clinical Medicine, Aalborg University, 9220 Aalborg, Denmark
* Correspondence: dwyew@hotmail.com

Supplementary Tables

Supplementary Table 1. Reagents used for turbidimetric analysis

Reagent	Properties	Main effect
Kaolin (Sigma-Aldrich, United Kingdom)	Hydrated aluminium silicate	Activates Factor XII
PPP reagent (Stago, France)	Mixture of phospholipids and tissue factor	Activates extrinsic pathway
PTT automate (Stago, France)	Mixture of cephalin (phospholipid) and a silica activator	Activates Factor XII
Thrombin (Sigma-Aldrich, United Kingdom)	Lyophilised human thrombin	Converts fibrinogen to fibrin

Supplementary Table 2. Terms relating to turbidimetric clotting analysis

Index	Description
Pre-plate reader time (T_{pre}), seconds	Time from combination of plasma and activation mix to the 1st measurement of the microplate reader. *Note that this value is different for each sample and column.*
Lag time (T_{lag}), seconds	Time taken from the 1st measurement of the microplate reader to when the absorbance becomes 0.005 units greater than the baseline value, accounting for the pre-plate reader time.
Peak absorbance (Abs_{peak}), units	Difference between the highest and lowest absorbance values.
Maximum absorbance (Abs_{max}), units	Calculated as the increase in absorbance from baseline to greatest absorbance at plateau. *Note that the plateau is often not a perfect horizontal line and the area of the plateau which is closest to horizontal is selected.*
Time to maximum absorbance (T_{max}), seconds	Time taken to achieve maximum absorbance, accounting for the pre-plate reader time.
Time to 50% peak absorbance ($T_{peak50\%}$), seconds	Time taken to achieve 50% peak absorbance, accounting for the pre-plate reader time.
Time to 50% clot lysis ($T_{lysis50\%}$), seconds	Time from peak absorbance to 50% peak absorbance.

Supplementary Table 3. Association between eGFR and fibrin clot properties in patients with atrial fibrillation

	Pearson's Correlation Coefficient (95% CI)	p value
Kaolin reagent		
T_{lag}	0.450 (0.132 to 0.684)	**0.008**
Abs_{peak}	-0.101 (-0.390 to 0.205)	0.518
T_{max}	0.385 (0.054 to 0.640)	**0.025**
$T_{peak50\%}$	0.409 (0.102 to 0.644)	**0.011**
$T_{lysis50\%}$	0.173 (-0.207 to 0.507)	0.371
PPP reagent		
T_{lag}	0.199 (-0.100 to 0.466)	0.466
Abs_{peak}	-0.069 (-0.352 to 0.226)	0.648
T_{max}	0.327 (0.037 to 0.566)	**0.028**
$T_{peak50\%}$	0.311 (0.020 to 0.554)	0.037
$T_{lysis50\%}$	0.205 (-0.095 to 0.470)	0.178
PTT reagent		
T_{lag}	0.073 (-0.222 to 0.356)	0.631
Abs_{peak}	0.031 (-0.262 to 0.318)	0.839
T_{max}	0.270 (-0.022 to 0.520)	0.069
$T_{peak50\%}$	0.030 (-0.262 to 0.318)	0.842
$T_{lysis50\%}$	-0.007 (-0.297 to 0.284)	0.964
Thrombin reagent		
T_{lag}	-0.196 (-0.460 to 0.100)	0.192
Abs_{peak}	-0.163 (-0.433 to 0.134)	0.279
T_{max}	-0.300 (-0.543 to -0.011)	**0.043**
$T_{peak50\%}$	-0.137 (-0.411 to 0.160)	0.365
$T_{lysis50\%}$	-0.153 (-0.427 to 0.147)	0.316
Ks	0.183 (-0.133 to 0.464)	0.253

Abs_{peak}, peak absorbance; CKD, chronic kidney disease; eGFR, estimated glomerular filtration rate; Ks, permeation constant; T_{lag}, lag time; $T_{lysis50\%}$, time to 50% clot lysis; T_{max}, time to maximum absorbance; $T_{peak50\%}$, time to 50% peak absorbance.

Supplementary Table 4. Effects of chronic kidney disease on fibrin clot properties in patients with atrial fibrillation

	CKD *vs.* non-CKD		
**Fibrin Clot Properties**	**Unadjusted Beta (95% CI)**	**Beta (95% CI)***	**p value**
Kaolin reagent			
T_{lag}	-589 (-930 to -249)	-778 (-1247 to -309)	**0.002**
Abs_{peak} (units, 340nm)	0.004 (-0.041 to 0.050)	-0.024 (-0.079 to 0.031)	0.384
T_{max}	-596 (-998 to -196)	-867 (-1423 to -310)	**0.004**
$T_{peak50\%}$	-569 (-980 to -158)	-853 (-1417 to -288)	**0.004**
$T_{lysis50\%}$	-110 (-403 to 184)	-10 (-428 to 408)	0.959
PPP reagent			
T_{lag}	-40 (-252 to 171)	-28 (-309 to 253)	0.841
Abs_{peak} (units, 340nm)	0.016 (-0.028 to 0.059)	0.007 (-0.050 to 0.065)	0.797
T_{max}	-163 (-466 to 139)	-169 (-572 to 235)	0.403
$T_{peak50\%}$	-182 (-470 to 106)	-170 (-554 to 214)	0.376
$T_{lysis50\%}$	-279 (-650 to 91)	-189 (-671 to 292)	0.431
PTT reagent			
T_{lag}	-6 (-29 to 18)	1 (-28 to 31)	0.921
Abs_{peak} (units, 340nm)	0.003 (-0.041 to 0.047)	-0.010 (-0.068 to 0.047)	0.718
T_{max}	-37 (-99 to 24)	-19 (-99 to 60)	0.624
$T_{peak50\%}$	1 (-31 to 33)	8 (-32 to 48)	0.691
$T_{lysis50\%}$	41 (-358 to 440)	210 (-289 to 709)	0.399
Thrombin reagent			
T_{lag}	58 (-77 to 194)	72 (-103 to 247)	0.411
Abs_{peak} (units, 340nm)	0.014 (-0.015 to 0.043)	-0.001 (-0.038 to 0.037)	0.974
T_{max}	196 (-61 to 452)	84 (-242 to 411)	0.604
$T_{peak50\%}$	28 (-204 to 259)	41 (-261 to 342)	0.787
$T_{lysis50\%}$	163 (-108 to 433)	292 (-50 to 634)	0.092
Ks (10^{-9} cm^2)	-3.87 (-10.16 to 2.42)	-2.03 (-10.14 to 6.09)	0.615

* Adjusted for age, diabetes mellitus, heart failure, statin use and haemoglobin level. Abs_{peak}, peak absorbance; CKD, chronic kidney disease; Ks, permeation constant; T_{lag}, lag time; $T_{lysis50\%}$, time to 50% clot lysis; T_{max}, time to maximum absorbance; $T_{peak50\%}$, time to 50% peak absorbance.

Supplementary Table 5. Association between eGFR and lipoprotein distribution in patients with atrial fibrillation

	**Pearson's Correlation Coefficient (95% CI)**	**p value**
Whole plasma		
Total cholesterol	0.326 (0.039 to 0.563)	**0.027**
LDL-C	0.478 (0.218 to 0.675)	**<0.001**
sdLDL	0.319 (0.032 to 0.558)	**0.031**
OxLDL	-0.055 (-0.340 to 0.239)	0.716
LDL subclass		
Total cholesterol	0.458 (0.193 to 0.660)	**0.001**
LDL-C	0.467 (0.204 to 0.667)	**0.001**
sdLDL	0.227 (-0.068 to 0.485)	0.129
OxLDL	-0.121 (-0.397 to 0.176)	0.424
lbLDL subclass		
Total cholesterol	0.494 (0.238 to 0.686)	**<0.001**
LDL-C	0.529 (0.282 to 0.710)	**<0.001**
sdLDL	0.376 (0.096 to 0.600)	**0.010**
OxLDL	-0.179 (-0.446 to 0.117)	0.233
sdLDL subclass		
Total cholesterol	0.337 (0.052 to 0.572)	**0.022**
LDL-C	0.267 (-0.026 to 0.517)	0.073
sdLDL	0.192 (-0.104 to 0.457)	0.201
OxLDL	-0.147 (-0.420 to 0.150)	0.329

lbLDL, large buoyant low-density lipoprotein; LDL-C, low-density lipoprotein cholesterol; OxLDL, oxidised low-density lipoprotein; sdLDL, small dense low-density lipoprotein.

Supplementary Table 6. Effects of chronic kidney disease on lipoprotein distribution in patients with atrial fibrillation

Lipoprotein Distribution	CKD *vs.* non-CKD Unadjusted Beta (95% CI)	CKD *vs.* non-CKD Beta (95% CI)*	p value
Whole plasma			
Total cholesterol	-0.78 (-1.34 to -0.21)	-0.33 (-1.01 to 0.34)	0.320
LDL-C	-0.87 (-1.37 to -0.37)	-0.44 (-0.99 to 0.10)	0.107
sdLDL	-0.22 (-0.43 to -0.01)	-0.15 (-0.40 to 0.10)	0.228
OxLDL (ng/mL)	0.83 (-15.85 to 17.51)	-2.67 (-23.34 to 18.00)	0.795
LDL subclass			
Total cholesterol	-1.62 (-2.59 to -0.64)	-0.74 (-1.87 to 0.39)	0.192
LDL-C	-1.53 (-2.47 to -0.58)	-0.55 (-1.62 to 0.51)	0.299
sdLDL	-1.67 (-0.44 to 0.11)	-0.11 (-0.46 to 0.24)	0.525
OxLDL (ng/mL)	2.78 (-5.72 to 11.29)	8.78 (-1.77 to 19.32)	0.100
lbLDL subclass			
Total cholesterol	-2.10 (-3.30 to -0.90)	-0.73 (-1.98 to 0.52)	0.246
LDL-C	-2.03 (-3.16 to -0.90)	-0.53 (-1.67 to 0.61)	0.354
sdLDL	-0.17 (-0.31 to -0.03)	-0.11 (-0.28 to 0.07)	0.238
OxLDL (ng/mL)	3.22 (-6.44 to 12.99)	5.73 (-6.98 to 18.44)	0.367
sdLDL subclass			
Total cholesterol	-0.97 (-1.95 to 0.01)	-0.43 (-1.59 to 0.73)	0.458
LDL-C	-0.65 (-1.53 to 0.22)	-0.25 (-1.34 to 0.85)	0.653
sdLDL	-0.23 (-0.70 to 0.24)	-0.16 (-0.75 to 0.44)	0.600
OxLDL (ng/mL)	2.11 (-6.37 to 10.58)	4.58 (-6.52 to 15.68)	0.409

* Adjusted for age, diabetes mellitus, heart failure, statin use and haemoglobin level. lbLDL, large buoyant low-density lipoprotein; LDL-C, low-density lipoprotein cholesterol; OxLDL, oxidised low-density lipoprotein; sdLDL, small dense low-density lipoprotein.

Supplementary Figure

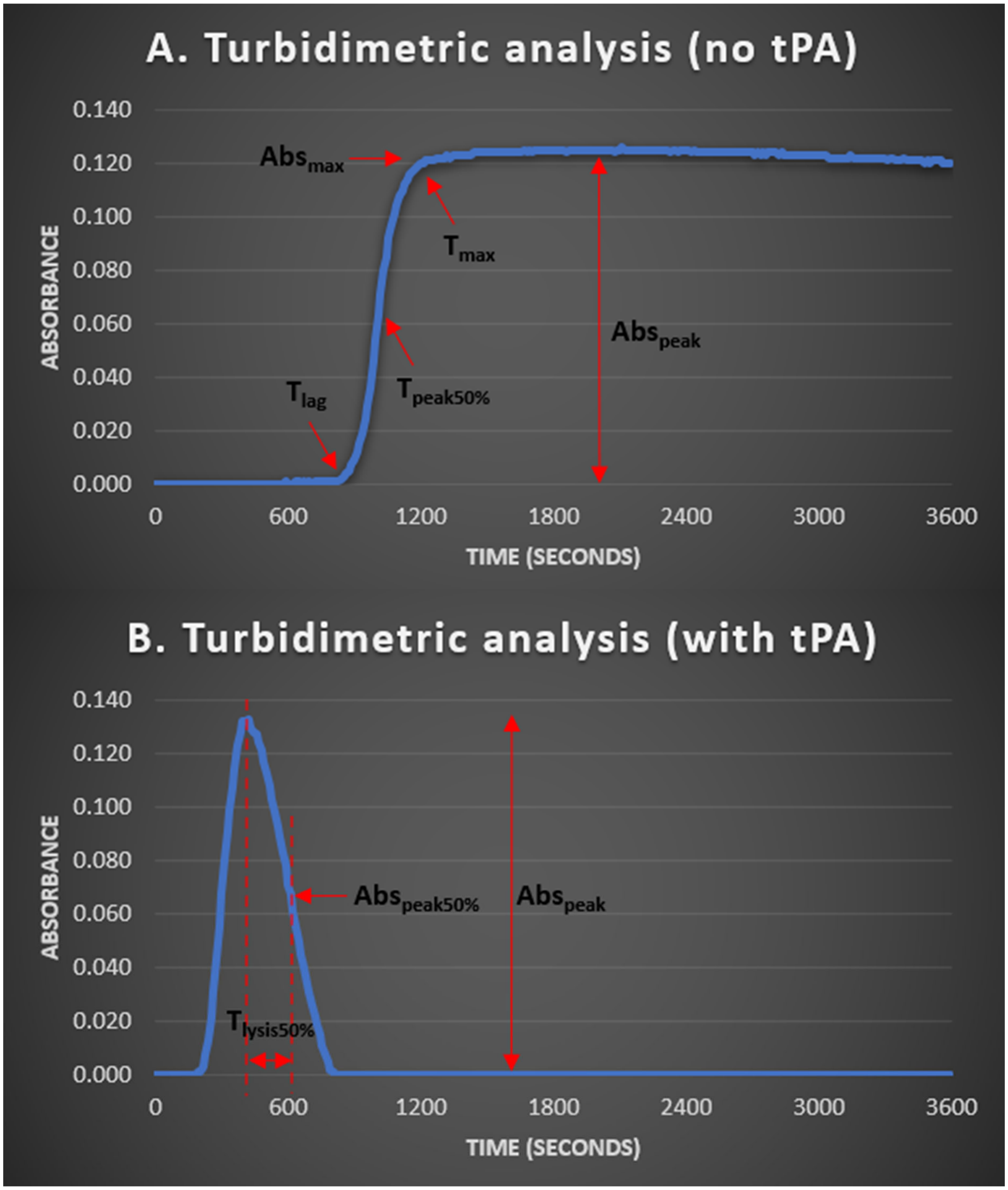

Supplementary Figure 1. Standard turbidimetric curves of clotting (A) and lysis (B) assays. Abs_{max}, maximum absorbance; Abs_{peak}, peak absorbance; T_{lag}, lag time; $T_{lysis50\%}$, time to 50% clot lysis; T_{max}, time to maximum absorbance; tPA, tissue plasminogen activator; $T_{peak50\%}$, time to 50% peak absorbance.
